# Bioremediation Potential of *Rhodococcus qingshengii* PM1 in Sodium Selenite-Contaminated Soil and Its Impact on Microbial Community Assembly

**DOI:** 10.3390/microorganisms12122458

**Published:** 2024-11-29

**Authors:** Mu Peng, Guangai Deng, Chongyang Hu, Xue Hou, Zhiyong Wang

**Affiliations:** 1 Hubei Key Laboratory of Biological Resources Protection and Utilization, Hubei Minzu University, Enshi 445000, China; pengmu1025@hotmail.com (M.P.); m18785717523@163.com (G.D.); hcy1784364088@outlook.com (C.H.); hx17320056301@163.com (X.H.); 2 College of Biological and Food Engineering, Hubei Minzu University, Enshi 445000, China

**Keywords:** selenium, *Rhodococcus qingshengii*, bioremediation, bacterial community assembly, stochastic processes

## Abstract

Soil microbial communities are particularly sensitive to selenium contamination, which has seriously affected the stability of soil ecological environment and function. In this study, we applied high-throughput 16S rRNA gene sequencing to examine the effects of low and high doses of sodium selenite and the selenite-degrading bacterium, *Rhodococcus qingshengii* PM1, on soil bacterial community composition, diversity, and assembly processes under controlled laboratory conditions. Our results indicated that sodium selenite and strain PM1 were key predictors of bacterial community structure in selenium-contaminated soils. Exposure to sodium selenite initially led to reductions in microbial diversity and a shift in dominant bacterial groups, particularly an increase in *Actinobacteria* and a decrease in *Acidobacteria*. Sodium selenite significantly reduced microbial diversity and simplified co-occurrence networks, whereas inoculation with strain PM1 partially reversed these effects by enhancing community complexity. Ecological modeling, including the normalized stochasticity ratio (NST) and Sloan’s neutral community model (NCM), suggested that stochastic processes predominated in the assembly of bacterial communities under selenium stress. Null model analysis further revealed that heterogeneous selection and drift were primary drivers of community turnover, with PM1 inoculation promoting species dispersal and buffering against the negative impacts of selenium. These findings shed light on microbial community assembly mechanisms under selenium contamination and highlight the potential of strain PM1 for the bioremediation of selenium-affected soils.

## 1. Introduction

Selenium (Se) is an essential trace element that plays a crucial role in the antioxidant defense systems and thyroid hormone metabolism of many organisms, including humans [1,2]. However, the narrow margin between its nutritional necessity and toxicity poses significant challenges in environmental management [3]. Selenium contamination is primarily a consequence of agricultural runoff, mining activities, and industrial discharges, which lead to elevated levels of selenium, particularly in its more soluble and toxic forms, such as selenite, in soils and aquatic systems [4,5]. High levels of selenite in soil or water can bioaccumulate in plants and animals, causing ecological disturbances and posing risks to human health through the food chain [4]. Therefore, determining how to eliminate selenite residues in soil or water has become an urgent problem. However, despite various proposed strategies to remediate selenite-contaminated soil [6,7,8], these physical and chemical methods have often proven largely time-consuming and uneconomical, underscoring the need for more efficient and feasible approaches. Microbial bioremediation has emerged as a promising alternative, as it leverages the natural capabilities of microbes to transform selenite into less toxic forms, such as elemental selenium (Se^0^) or organic selenium compounds (e.g., selenomethionine), thereby reducing its toxicity and mobility [9]. Selenite-reducing bacteria not only detoxify selenium but also influence its bioavailability and ecological impact, playing a pivotal role in the biogeochemical cycling of selenium [10,11]. Various bacterial genera [12], including *Bacillus* [13], *Stenotrophomonas* [14], *Chitinophaga* and *Comamonas* [15], have demonstrated selenite-reducing capabilities, enhancing our understanding of microbial detoxification pathways. However, research on *Rhodococcus qingshengii* is limited, with only one study isolating this bacterium from selenium-rich mines [16]. This gap suggests an untapped potential for discovering novel selenite-reducing mechanisms in underexplored bacterial taxa, including R. qingshengii, to develop more effective selenium bioremediation strategies.

Heavy metal pollution has become a prominent issue in soil contamination in China, significantly impacting soil ecosystem health and functional stability [17]. As an essential component of soil, microbial communities serve as effective indicators of the ecological effects of heavy metal pollution [18]. Under heavy metal stress, microbial communities adapt to polluted environments through shifts in diversity, community structure, and functionality [19]. For example, studies have found that heavy metals selectively inhibit the growth of sensitive microorganisms, allowing resistant strains to dominate [20]. While this change enhances the tolerance of certain strains within the soil, it may also reduce overall biodiversity, potentially compromising soil ecological stability and self-restoration capacity [21]. Different types of microorganisms respond to heavy metals in varying ways. Beyond growth inhibition, heavy metals can impact microorganisms through oxidative stress, DNA damage, and other mechanisms that further affect gene expression and metabolic activity [22]. Heavy metal pollution also disrupts nutrient cycling processes, such as those involving carbon and nitrogen [23]. This imbalance in nutrient cycling further weakens the health and resilience of soil ecosystems. Therefore, understanding the impact of heavy metal pollution on microbial communities is essential for developing effective bioremediation strategies. By utilizing resistant microorganisms to remove contaminants and restore soil ecological functions while maintaining ecological balance, we can provide a scientific basis for soil remediation efforts.

Microbial community assembly is shaped by deterministic processes (such as environmental selection) and stochastic processes (such as drift and dispersal limitation) [24]. These processes interact within various ecosystems to maintain microbial diversity and stability. Community assembly, a core issue in microbial ecology, has been extensively studied in forest, marine, and soil ecosystems [25,26,27]. Neutral theory assumes that microbial community dynamics are governed by stochastic processes, including ecological drift, random birth and death events, and dispersal limitations [28]. Conversely, traditional niche-based theory hypothesizes that community assembly is primarily due to deterministic processes, where environmental filtering (e.g., energy, carbon availability, and moisture content) and microbial interactions (e.g., competition, predation, and symbiosis) play critical roles in defining unique niches [29]. The relative contribution of these processes to microbial community assembly varies depending on environmental conditions and host types [30]. Research on bacterial community structures in response to pollutants has shown that both deterministic and stochastic processes contribute to community assembly under stress conditions [18,31,32]. Microbial communities adapted to heavy metal stress through complex interactions, which tend to favor cooperation over competitive behaviors. However, the specific dynamics of community assembly in selenite-contaminated soils have not been reported thus far.

While microbial remediation of metal-contaminated soils has been widely studied, there is limited insight into how specific bacterial strains, such as *R. qingshengii*, influence microbial community composition and ecological resilience under selenium stress. In this study, we used high-throughput 16S rRNA gene sequencing and bioinformatics analysis to investigate the effects of sodium selenite and the selenite-degrading strain *R. qingshengii* PM1 on soil bacterial communities under controlled laboratory conditions. Our primary objectives were: (1) to determine the ecological impacts of sodium selenite on soil microbial community composition, (2) to evaluate the role of strain PM1 in facilitating microbial community recovery, and (3) to quantify microbial community assembly processes using four ecological models. We hypothesize that inoculation with strain PM1 will not only reduce selenium contamination but also promote soil ecological stability by increasing microbial α- and β-diversity and enhancing network complexity. This study addresses critical gaps in understanding microbial community assembly in selenium-contaminated environments and assesses the bioremediation potential of R. qingshengii PM1, providing a foundation for developing sustainable pollution management strategies in selenium-affected ecosystems.

## 2. Material and Methods

### 2.1. Soil Collection and Treatments

For the pot experiment, soil with a near-neutral pH (typical yellow-brown soil, pH 7.8) was collected from the 0–20 cm layer in the flower garden of Hubei Minzu University, Enshi, Hubei Province, China. After sampling, plant roots, gravel, and other debris were removed. The soil samples were then air-dried naturally and passed through a 2 mm sieve to ensure soil uniformity consistency for experimental use. Two portions of 200 g homogenized soil (dry weight) were mixed thoroughly with 50 mL sterile deionized water containing 46.3 μL and 231.4 μL of 500 mM sodium selenite, respectively. The final concentrations of selenite were 20 and 100 mg/kg. Soil without added sodium selenite was treated with 50 mL of sterile deionized water.

*Rhodococcus qingshengii* PM1, a highly selenite-resistant strain, was isolated from a highly selenium-rich mine, Enshi City, Hubei Province, China [16]. The strain has the highest selenite reduction capability at 100 mM, indicating its potential for bioremediation of selenium-contaminated environments [33]. Strain PM1 was cultured in Lysogeny Broth (LB) medium for 48 h, and the cells were collected by centrifugation, washed twice, and resuspended in PBS buffer solutions (phosphate-buffered saline). This cell suspension was then evenly mixed with the soil to a final concentration of approximately 1 × 10^8^ CFU (colony-forming units)/g dry soil. Soil without inoculation was treated with PBS buffer.

Finally, a total of six treatments were included in our experiment based on the doses of sodium selenite and strain PM1, such as (i) non-treatment soil (NO), (ii) low-dose selenium-contaminated soil (LO), (iii) high-dose selenium-contaminated soil (HI), (iv) non-treatment soil + strain PM1 (PM1), (v) low-dose selenium-contaminated soil + strain PM1 (LO + PM1), (vi) high-dose selenium-contaminated soil + strain PM1 (HI + PM1). All pots were cultivated in a climate-controlled chamber with a 12 h daytime period at 25 °C and 4000 l× and a 12 h night period at 20 °C with no light. Soil moisture was maintained at approximately 25% (*w*/*w*), and water loss was replenished daily by weighing the pots. Soil sampling was conducted at 1, 10, 20, and 30 days of the experiment, and samples were stored at −80 °C for the next research. All treatments were carried out in triplicate. The soil physicochemical properties were measured as follows: soil organic carbon (SOC) was assessed using a total carbon analyzer (Multi N/C 2100, Jena Analytic, Jena, Germany), while total nitrogen (TN) was measured with a Vario MACRO cube elemental analyzer (Elementar, Hanau, Germany). Total phosphorus (TP) was determined using H_2_SO_4_-HClO_4_ digestion and total potassium (TK) content was quantified by NaOH fusion and flame photometry method. Six soil enzyme indexes (fluorescein diacetate hydrolase, β-glucosidase, urease, phenol oxidase, nitrate reductase, peroxidase) were performed according to the manufacturer’s protocol (Sangon Biotech Co., Ltd., Shanghai, China). For residual sodium selenite analysis, the supernatant of soil was filtered through Millipore filters (0.22 µm), and the concentration of residual selenite was quantified using inductively coupled plasma optical emission spectrometry (ICP-OES, Thermo Fisher Scientific, Waltham, MA, USA).

### 2.2. DNA Extraction, Library Construction, and Sequencing

A total of 72 soil samples were collected, and total DNA was extracted using a commercial soil DNA extraction kit (Power Soil DNA Isolation Kit, Qiagen, Hilden, Germany) following the manufacturer’s instructions from a 0.1 g soil sample. The V3-V4 region of the 16S rRNA gene was amplified using specific primers (338F/806R) with overhang adapters for Illumina MiSeq sequencing (Majorbio Biotech Co., Ltd., Shanghai, China). Illumina MiSeq platform was used to sequence the 16S rRNA amplicons. Sequencing data were processed using the appropriate bioinformatics pipelines to generate high-quality reads for downstream analysis.

### 2.3. Statistical Analysis

Sequencing raw data were analyzed using QIIME2 (24 December 2023, https://qiime2.org/) and then assembled, filtered, and clustered into operational taxonomic units (OTUs) with 97% sequence similarity as the cutoff point using UPARSE software (version 7.0.1090, 24 December 2023, http://drive5.com/uparse/). After filtering, the reads were subsampled to 33599 reads per sample. OTUs classified as chimeras, chloroplast, or mitochondria were removed using the MOTHUR program (version 1.44.0) [34]. The sequences were assigned using the RDP classifier (Release 11.5) for taxonomic assignment [35]. Alpha diversity (e.g., Shannon, Chao1 indices, phylogenetic diversity) and beta diversity (e.g., Bray–Curtis dissimilarity) were calculated to assess microbial community diversity and composition. Principal coordinates analysis (PCoA) was used to visualize differences in microbial communities among samples. Statistical tests, such as ANOVA (analysis of variance) and PERMANOVA (permutational multivariate analysis of variance), were conducted using the “vegan” package in R (version 4.3.3) to determine the significance of observed differences in microbial community composition. For ANOVA, Tukey’s post hoc test was used to compare means between groups, and significance was considered at *p* < 0.05. To test the significance of the samples, three different non-parametric statistical methods (Adonis, ANOSIM, and MRPP) were used to determine the overall differences in bacterial communities [36]. Redundant analysis (RDA) was carried out to illustrate the effects of soil physicochemical properties on soil microbial diversity. Structural equation models (SEMs) were established to identify the direct and indirect effects of Se and strain PM1 on soil properties and microbial diversity. Random forest analysis was performed to identify the key soil physicochemical properties and enzyme activities influencing microbial community structure.

### 2.4. Co-Occurrence Network Analysis and Community Assembly

Co-occurrence network analysis was performed to explore the relationships between different microbial taxa using a phylogeny molecular ecological network analysis pipeline (pMENA) [37]. Only the OTUs existing in more than 10 samples were retained for the subsequent analysis. OTU tables were filtered to remove low-abundance taxa to reduce noise. Relative abundances were used to standardize the data. Network topology parameters, including node degree, betweenness centrality, and modularity, were calculated to identify key taxa (hubs) and network modules. The network was visualized using Gephi [38]. Microbial network stability, such as vulnerability and robustness, were calculated using the code in the published paper of Yuan et al. [39].

In order to quantify the relative importance of deterministic and stochastic processes in community assembly, four distinct microbial assembly models were employed to investigate the community assembly mechanisms within a given ecosystem. First, the normalized stochasticity ratio (NST) was used to estimate the relative roles of these processes, with NST values indicating stochastic (>50%) or deterministic (<50%) dominance in community structure [40]. Second, the neutral community model (NCM) assessed neutrality in community dynamics, comparing observed patterns with those expected under neutral theory, where *Nm* represents dispersal estimates, *N* is metacommunity size, and *m* is immigration rate [41]. The parameter *R*^2^ represents the overall fit to the neutral model. To ensure robust results, a 95% confidence interval was calculated using 1000 bootstrap replicates. Thirdly, the β-nearest taxon index (β-NTI) was used to evaluate phylogenetic turnover, indicating whether community changes are driven by deterministic or stochastic forces. Specifically, β-NTI values greater than +2 or less than −2 suggest deterministic processes, while values between −2 and +2 indicate stochastic influences [42]. Additionally, the Bray–Curtis-based Raup–Crick metric (RCbray) quantified dissimilarities in community assembly, with values above +0.95 indicating dispersal limitation, values below −0.95 suggesting homogenizing dispersal, and values between −0.95 and +0.95 associated with drift [43]. Lastly, the iCAMP model (infer community assembly mechanisms by phylogenetic-bin-based null model analysis) classified assembly processes using the beta net relatedness index (βNRI) and Raup–Crick metric (RC), identifying deterministic processes [homogeneous selection (HoS, βNRI > +2) and heterogeneous selection (HeS, βNRI < −2)] and stochastic processes [dispersal limitation (DL, βNRI between −2 and +2, RC > +0.95), homogenizing dispersal (HD, βNRI between −2 and +2, RC < −0.95), and drift (DR, βNRI between −2 and +2, RC between −0.95 and +0.95)] [44]. Together, these four models provided a comprehensive framework for understanding the complex interplay of stochastic and deterministic forces shaping microbial community assembly.

### 2.5. Data Availability

All the bacterial raw sequences have been deposited to GenBank Short Read Archive (PRJNA1136399).

## 3. Results

### 3.1. Effects of Sodium Selenite and Strain PM1 on Soil Physicochemical Properties

Soil physicochemical properties are crucial for plant growth, ecosystem functions, and soil quality. Table 1 shows that soil organic carbon (SOC) decreased significantly in the early stages (1 d and 10 d) but then increased by day 20 with sodium selenite treatment, remaining higher than in the control group (NO) by day 30. Total nitrogen (TN) in the HI group increased significantly in the early stages and remained higher than that in the NO group throughout the experiment, while total potassium (TK) content was higher in the early stages but decreased significantly by day 30. Total phosphorus (TP) initially increased, decreased by day 20, and then recovered to baseline by day 30. The addition of strain PM1 (HI + PM1) significantly mitigated these effects, resulting in higher SOC in the early stages, sustained TN levels, and increased TP by day 30 compared to the HI group. This indicates that strain PM1 effectively alleviates the adverse effects of sodium selenite on soil microorganisms and enhances soil remediation capabilities, particularly in the later stages of treatment.

Soil enzymes, crucial biocatalysts derived from plant roots, residues, and microorganisms, are vital for biochemical reactions and soil health. Sodium selenite inhibited fluorescein diacetate hydrolase (FDA) activity, especially at higher concentrations, but strain PM1 boosted FDA activity in sodium selenite-treated groups (Table 1). Sodium selenite affected peroxidase (POD) activity early, with PM1 further inhibiting POD under these conditions. Sodium selenite impacted β-glucosidase (GC) activity early, but strain PM1 mitigated these effects initially. Sodium selenite influenced nitrate reductase (NR) activity, particularly at lower concentrations, but PM1 alleviated this effect. Soil polyphenol oxidase (PPO) activity increased due to PM1, particularly on the first day, due to its enhancement of microbial activity. Finally, soil urease (UE) activity was inhibited by sodium selenite, but strain PM1 mitigated this inhibition. Overall, sodium selenite negatively impacted soil enzyme activities, but strain PM1 alleviated these effects, enhancing soil resilience against sodium selenite stress (Table 1).

### 3.2. Dynamics of Residual Sodium Selenite in Each Soil Treatment

**Figure 1:**
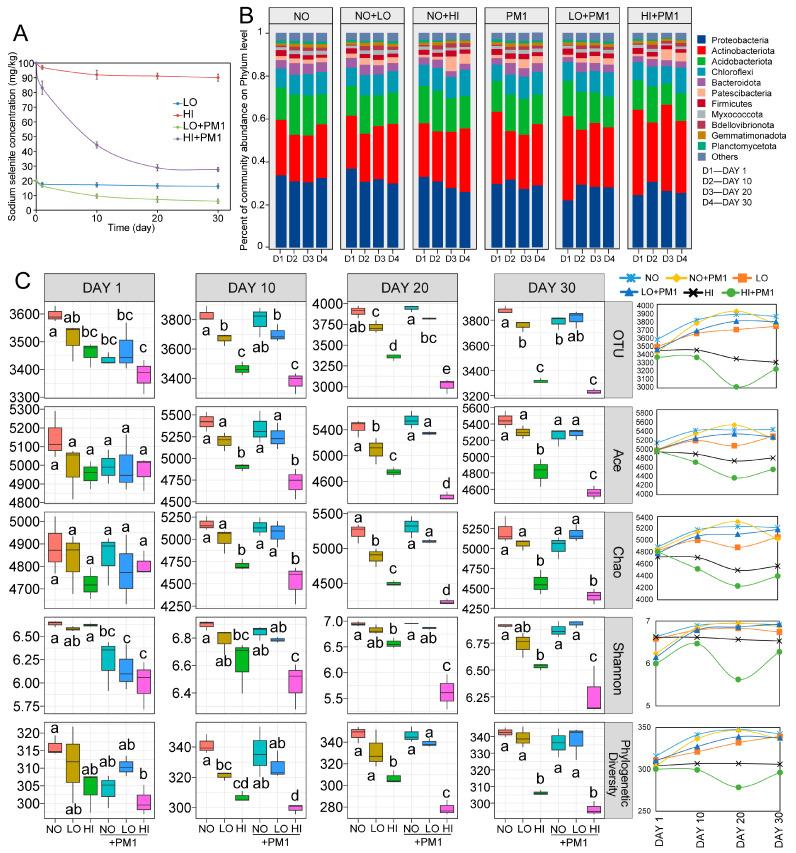
Dynamic changes in sodium selenite concentration, microbial community composition, and alpha diversity in soils under different selenium contamination treatments. (**A**) Changes in sodium selenite in each selenium-contaminated treatment during the incubation period. (**B**) Phylum-level relative abundance of bacterial communities across treatment groups at different time points (Day 1, Day 10, Day 20, and Day 30). (**C**) Alpha diversity metrics, including OTU, Chao1, Shannon diversity index, and phylogenetic diversity across treatment groups and incubation times. Significant differences between treatments are indicated by different letters above the box plots (*p* < 0.05). The right-side graphs illustrate changes in each alpha diversity index over time, showing the impact of sodium selenite and strain PM1 on microbial diversity. NO: non-treatment soil, LO: low-dose selenium-contaminated soil, HI: high-dose selenium-contaminated soil, PM1: non-treatment soil + strain PM1, LO + PM1: low-dose selenium-contaminated soil + strain PM1, HI + PM1: high-dose selenium-contaminated soil + strain PM1.

The reduction of sodium selenite in soil reflects microbial metabolic activity. In soils treated with strain PM1, sodium selenite reduction reached 63.39% to 71.14% after 20 days (LO + PM1, HI + PM1), compared to lower reduction rates of 8.84% to 17.37% in untreated soils (LO, HI) (Figure 1A). Extending the incubation period to 30 days showed minimal additional reduction. These results indicate that strain PM1 significantly enhances sodium selenite degradation at both low and high contamination levels. Overall, strain PM1 demonstrates robust bioremediation potential for sodium selenite, supporting findings from previous liquid culture studies and underscoring its practical suitability for soil remediation efforts.

### 3.3. Effects of Sodium Selenite and Strain PM1 on Soil Bacterial Community Composition and Alpha Diversity

After filtering out non-target sequences (i.e., chloroplasts, mitochondria, archaea, and unassigned), a total of 4,842,944 high-quality bacterial sequences were generated across 72 samples, with an average of 67,263 sequences per sample (Table S1). Overall, 24,459 bacterial OTUs were identified at 97% sequence similarity across all samples. The effects of sodium selenite and strain PM1 on soil microbial communities were primarily reflected in differences in community composition at the phylum level across treatments. Results showed that, although the overall composition of soil microbial communities was largely similar across treatments, the relative abundance of dominant bacterial phyla varied significantly (Figure 1B). The dominant bacterial phyla in all samples primarily included *Proteobacteria*, *Actinobacteria*, and *Acidobacteria*, accounting for 22.04–38.47%, 21.83–41.95%, and 8.41–20.41% of the total sequences, respectively. In the early stages of incubation, compared to the control group (NO), low doses of sodium selenite (LO) increased the relative abundance of *Proteobacteria* (33.18% to 38.47%) and decreased the relative abundance of *Actinobacteria* (27.27% to 20.79%), while high concentrations of sodium selenite (HI) had a less noticeable effect (33.18% to 35.17% and 27.27% to 24.78%).

Interestingly, inoculation with strain PM1 led to significant changes in the phylum-level composition of soil bacterial communities under different doses of sodium selenite. In the early stages of incubation, strain PM1 significantly increased the relative abundance of *Proteobacteria* but inhibited the relative abundance of *Actinobacteria*. As incubation progressed, high doses of sodium selenite inhibited the proportion of *Proteobacteria* (35.17% to 26.06%), while the relative abundance of *Proteobacteria* stabilized in the mid to late stages after inoculation with strain PM1 (Figure 1B). Compared to the HI group, inoculation with PM1 (HI + PM1) increased the relative abundance of *Actinobacteria* (Day 1: 24.78% to 38.49%, Day 10: 23.92% to 31.48%, Day 20: 28.28% to 41.95%, and Day 30: 31.15% to 35.01%).

After subsampling, Good’s coverage of soil bacterial communities ranged from 95.5% to 96.6%, indicating sufficient sampling depth for α diversity analysis (Figure S1). As expected, in the early stages of incubation, α diversity decreased with increasing doses of sodium selenite. After 20 days, α diversity remained at a low level under high-dose sodium selenite treatments (HI, HI + PM1) (Figure 1C). However, compared to NO, the low-dose sodium selenite treatments (LO, LO + PM1) nearly returned to normal, suggesting that low-dose sodium selenite had no significant effect on soil α diversity. In the HI + PM1 group, α diversity showed a more pronounced decrease in the early stages of inoculation compared to the HI group but began to increase after 20 days, indicating that strain PM1 initially acted as an invasive species, impacting the native soil community before beginning to facilitate soil remediation. Consistent with the community composition analysis results (Figure 1C), the negative impact of high-dose sodium selenite on α diversity gradually increased during the incubation period. A similar trend was observed in the strain inoculation group (HI + PM1, Figure 1C). While strain PM1 briefly reduced α diversity in the early stages of inoculation, this negative effect gradually decreased after 20 days. This indicates that strain PM1 mitigated the impact of high-dose sodium selenite on α diversity (HI + PM1, Figure 1C). These results suggest that the effect of sodium selenite on soil bacterial community composition intensified over time, while strain PM1’s degradation capability mitigated sodium selenite’s ecological impact on soil bacterial communities.

### 3.4. Effects of Sodium Selenite and Strain PM1 on Soil Bacterial Beta Diversity

**Figure 2:**
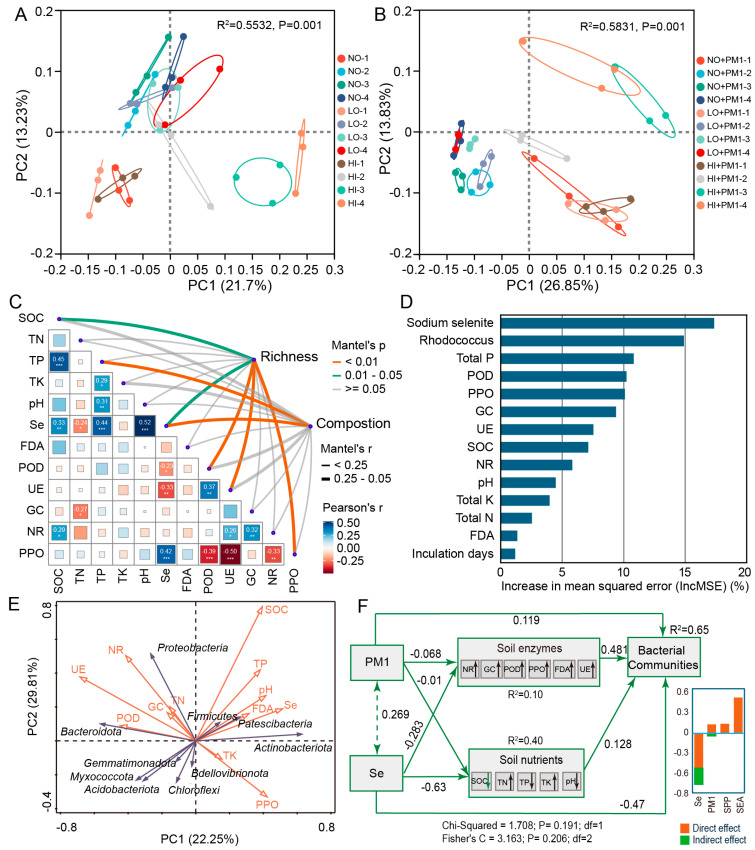
Effects of sodium selenite and strain PM1 on soil bacterial beta diversity. (**A**,**B**) Principal coordinates analysis (PCoA) plots showing bacterial community structure. (**A**) Comparison of community structure across different treatments. (**B**) Community structure in treatments with PM1 inoculation. (**C**) Mantel test analysis showing the relationship between soil physicochemical properties and β-diversity of the bacterial community. Thicker lines indicate larger Mantel’s r values and the line color represents different significance levels. Bacterial richness is represented by the Chao index, and diversity is indicated by the Shannon index. Soil nutrients are represented by soil properties and enzyme activities. (**D**) Random forest analysis identifying key factors influencing bacterial community composition. (**E**) Redundancy Analysis (RDA) illustrating the effects of soil physicochemical properties on soil microbial diversity. (**F**) Structural equation models (SEMs) depicting the direct and indirect effects of sodium selenite and strain PM1 on soil properties, enzymes, and microbial community diversity. NO: non-treatment soil, LO: low-dose selenium-contaminated soil, HI: high-dose selenium-contaminated soil, PM1: non-treatment soil + strain PM1, LO + PM1: low-dose selenium-contaminated soil + strain PM1, HI + PM1: high-dose selenium-contaminated soil + strain PM1. *** *p* < 0.001; ** *p* < 0.01; * *p* < 0.05.

PCoA analysis showed that the beta diversity of soil bacteria was primarily influenced by sodium selenite, strain PM1, and incubation time (Figure 2A,B). In the early stages of incubation, the sample points clustered closely together, indicating that sodium selenite and strain PM1 initially had an insignificant effect on soil bacterial diversity. However, as the incubation period progressed, the sample points became more dispersed, suggesting that the influence of sodium selenite, strain PM1, and incubation time on bacterial diversity became more pronounced (Figure 2A,B). Notably, after 10 days of incubation, strain PM1 further enhanced the impact of sodium selenite on soil bacterial diversity (Figure 2B). Statistical significance tests confirmed that sodium selenite, strain PM1, and incubation time were significant factors affecting soil bacterial diversity (Table 2). Overall, the degradation of sodium selenite by strain PM1 significantly enhanced its ecological impact on soil bacteria, particularly under high-dose sodium selenite conditions.

The Mantel test was used to analyze the correlation between environmental variables and bacterial community structure in selenium-contaminated soils. The results indicated that sodium selenite concentration was the primary environmental factor driving changes in bacterial community composition (Figure 2C). Additionally, SOC, TP, POD, UE, and PPO played positive roles in shaping the local bacterial communities, suggesting that changes in soil properties disrupted bacterial activity and promoted community restructuring. Random forest analysis identified the roles of soil physicochemical properties in bacterial community assembly, showing that sodium selenite and strain PM1 were key predictors of soil bacterial community structure (Figure 2D). RDA analysis revealed that sodium selenite concentration was strongly positively correlated with soil nutrients such as SOC, pH, and TP, indicating that the addition of sodium selenite may increase the availability of bioavailable nutrients in the soil (Figure 2E). In particular, high sodium selenite concentrations strongly correlated with certain bacterial communities, with salt-tolerant, selenium-resistant bacteria, such as *Actinobacteria* and *Proteobacteria*, becoming more dominant under selenium stress.

To better integrate the complex interactions between soil variables, bacterial communities, and sodium selenite, a structural equation model (SEM) was used. The latent variables (shown as blue rectangles in Figure 2F) successfully captured the relationships among soil nutrients, bacterial communities, and sodium selenite availability. The results indicated that soil sodium selenite availability and strain PM1 had significant direct effects on bacterial communities. This integrated analysis revealed that both abiotic factors (sodium selenite and soil nutrients) and biotic factors (strain PM1 and enzymes) influenced microbial community structure in selenium-contaminated soils. Selenium emerged as a critical driver of microbial diversity changes, while soil composition positively affected selenium availability.

### 3.5. Effects of Sodium Selenite and Strain PM1 on Soil Bacterial Co-Occurrence Networks

Co-occurrence networks are essential for understanding the complex interactions and relationships within microbial communities, as they can reveal underlying ecological processes and community dynamics. Based on Table 2, significant differences were observed among bacterial communities across treatments. Thus, six bacterial co-occurrence networks were constructed based on the dose of sodium selenite and inoculation with strain PM1. All six networks were non-random and exhibited topological features typical of complex systems, such as scale-free structure, small-world characteristics, and modularity (Table 3).

**Figure 3:**
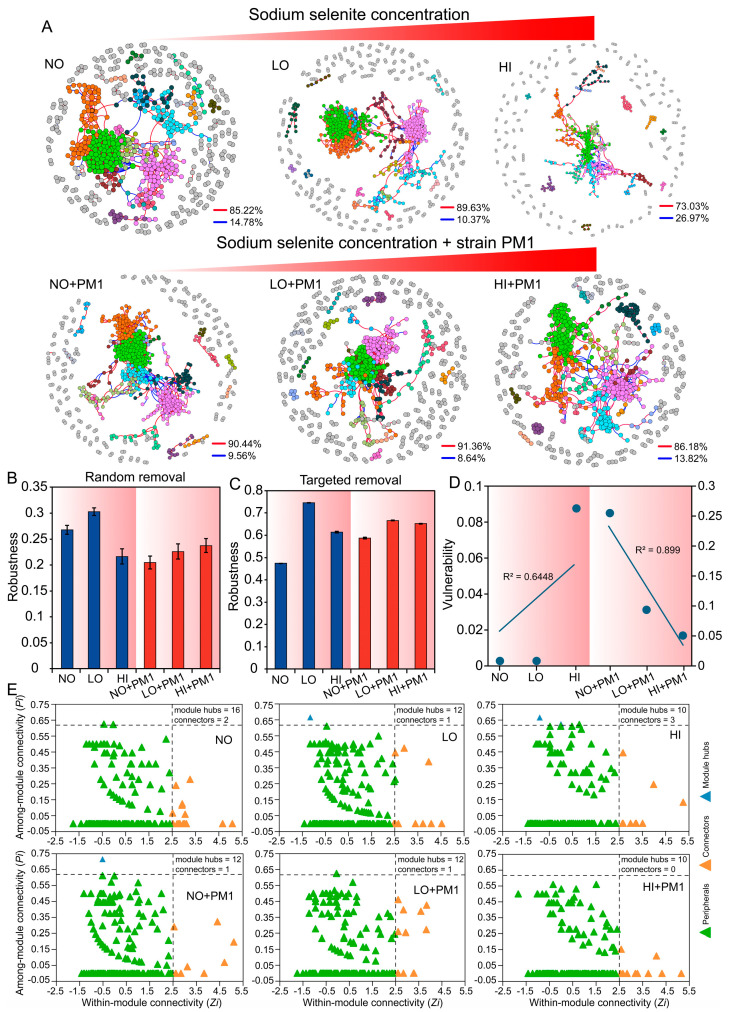
Co-occurrence network analysis of soil microbial community based on Pearson’s correlation analysis among OTUs. (**A**) Blue and red lines represent significant negative and positive correlations, respectively. The nodes indicate the OTUs, and the color of the node represents the model hub. Robustness is calculated as the proportion of remaining species in the community after randomly removing 50% of the nodes (**B**) or targeted hubs (**C**), while vulnerability (**D**) is determined by the highest node vulnerability in each network. (**E**) Putative keystone taxa in the different networks based on *Pi* and *Zi*. The solid triangle represents an OTU. NO: non-treatment soil, LO: low-dose selenium-contaminated soil, HI: high-dose selenium-contaminated soil, PM1: non-treatment soil + strain PM1, LO + PM1: low-dose selenium-contaminated soil + strain PM1, HI + PM1: high-dose selenium-contaminated soil + strain PM1.

Co-occurrence network analysis showed that sodium selenite and strain PM1 significantly affected the topological features of bacterial networks. As the concentration of sodium selenite increased, network size (total nodes), total links, average degree, average clustering coefficient, and centralization betweenness all decreased, indicating that the complexity of microbial networks decreased sharply and interspecies connections became simplified with increasing sodium selenite concentrations (Figure 3A and Table 3). Modularity and the number of modules, however, showed an increasing trend with increasing sodium selenite concentrations. A similar trend was observed in the strain-inoculated groups. Positive correlations among nodes were much higher than negative correlations. Furthermore, compared to the LO and HI treatment groups, the complexity of bacterial networks significantly increased in the strain-inoculated groups (LO + PM1, HI + PM1), suggesting that strain PM1 contributes to soil remediation to some extent (Figure 3A and Table 3).

The stability of soil microbial networks was tested by simulating the effect of species extinction on average degree and natural connectivity through random node removal. The results showed that as sodium selenite concentration increased, the robustness of the networks initially increased and then decreased whether nodes were removed randomly or in a targeted manner (Figure 3B,C). These findings indicate that high doses of sodium selenite led to more fragile network structures. In the strain-inoculated groups, network robustness increased with rising sodium selenite concentrations, suggesting that strain PM1 enhances bacterial network complexity to a certain extent. Additionally, network vulnerability was significantly higher in the PM1-inoculated groups (0.051–0.2551) compared to the sodium selenite-treated groups (0.0001–0.087), indicating higher robustness in sodium selenite-treated networks (Figure 3D). Subsequently, potential keystone taxa (peripheral nodes, connectors, module hubs, and network hubs) were identified in the network graphs. The results showed fewer keystone OTUs in the sodium selenite and PM1 treatment groups than in the NO group (Figure 3E), with many potential keystone OTUs belonging to the phylum *Proteobacteria* (Table S2). The topological features of bacterial network complexity exhibited both positive and negative correlations with the concentration of sodium selenite, indicating that the effect on microbial network complexity is dose-dependent. For example, total nodes, total links, and average degree were negatively correlated with sodium selenite concentration, while modularity showed a strong positive correlation (*p* < 0.01) (Figure S2). However, no significant correlations were found between network complexity and stability indices under these treatments. In summary, increasing sodium selenite concentrations significantly reduced the complexity and stability of microbial ecological networks, while strain PM1 mitigated these effects and enhanced soil bioremediation capabilities.

### 3.6. Ecological Processes Governing Bacterial Community Assembly

**Figure 4:**
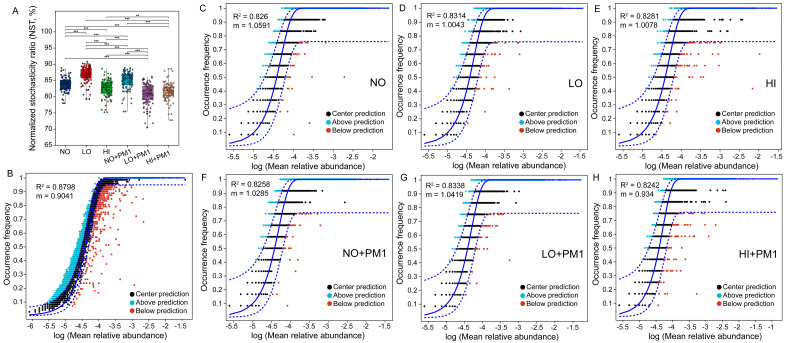
The normalized stochasticity ratio (NST) (**A**) and Sloan’s neutral community model (NCM) (**B**–**H**) were used to assess the soil bacterial community assembly process. The blue solid line represents the fit of the neutral model, and the upper and lower blue dashed lines represent the 95% confidence interval predicted by the model. NO: non-treatment soil, LO: low-dose selenium-contaminated soil, HI: high-dose selenium-contaminated soil, PM1: non-treatment soil + strain PM1, LO + PM1: low-dose selenium-contaminated soil + strain PM1, HI + PM1: high-dose selenium-contaminated soil + strain PM1. *** *p* < 0.001; ** *p* < 0.01.

To elucidate the effects of sodium selenite and strain PM1 on bacterial community assembly, four ecological models were applied. Firstly, the normalized stochasticity ratio (NST) was used to quantify the roles of deterministic and stochastic processes in microbial community assembly (Figure 4A, Table S3). The NST values for all bacterial communities exceeded the 50% threshold, ranging from 80.80% to 86.78%, indicating that stochastic processes dominated over deterministic processes in the microbial communities treated with sodium selenite and strain PM1. Furthermore, as sodium selenite concentration increased, the contribution of stochastic processes initially increased and then decreased. However, in the strain PM1 inoculation group, the contribution of stochastic processes gradually declined. Significant differences in the NST values were observed across treatment groups, except between LO + PM1 and HI + PM1 (*p* > 0.05) (Figure 4A).

To explore the impact of sodium selenite and strain PM1 on the neutral processes of community assembly, Sloan’s neutral community model (NCM) was employed. The frequency of bacterial OTUs within all bacterial communities showed a high degree of fit with the neutral model, with most OTUs falling within the 95% confidence interval predicted by the NCM (R² = 0.8242–0.8798, m = 0.9041–1.0591) (Figure 4B–H). This indicates that stochastic processes played a more crucial role than deterministic processes in bacterial community assembly. The migration rates of bacterial communities (*m*) in sodium selenite-treated soils (1.0043–1.0078) (Figure 4D,E) were lower than those in the control group (1.0591) (Figure 4C), suggesting that species dispersal was more restricted in sodium selenite-treated soils. Similarly, the *Nm* values in sodium selenite-treated groups (33,744–33,862) were significantly lower than those in the control group (35,584), indicating that sodium selenite treatment inhibited species dispersal and reduced OTU occurrence frequency in the soil (Table S4). After inoculation with strain PM1, both the migration rate and *Nm* showed a decreasing trend. Compared to the LO group, the R², *m*, and *Nm* values for LO + PM1 were higher, indicating that inoculation with strain PM1 alleviated the restrictions on species dispersal.

**Figure 5:**
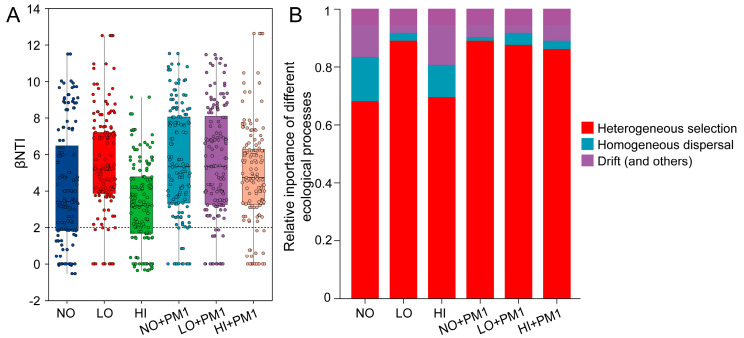
Distribution of beta nearest taxon index (β-NTI) among different samples (**A**). The proportion of heterogeneous selection, homogeneous dispersal and drift in the microbial assembly process (**B**). NO: non-treatment soil, LO: low-dose selenium-contaminated soil, HI: high-dose selenium-contaminated soil, PM1: non-treatment soil + strain PM1, LO + PM1: low-dose selenium-contaminated soil + strain PM1, HI + PM1: high-dose selenium-contaminated soil + strain PM1.

Additionally, a null model was used to quantify the relative influence of stochastic and deterministic forces in shaping bacterial community assembly. Since the beta nearest taxon index (β-NTI) values for all samples ranged between 3.426 and 5.3401, deterministic processes were identified as the primary factors influencing bacterial community assembly (Figure 5A). By combining weighted β-NTI and Bray–Curtis-based Raup–Crick (RCbray) metrics, we estimated the relative contributions of different assembly processes. Null model analysis assessed the ecological processes governing community composition following sodium selenite treatment and strain PM1 inoculation. When taxonomic information was not considered, heterogeneous selection emerged as the dominant factor affecting bacterial community assembly (β-NTI > 2, Figure 5B), explaining 68.06% to 88.89% of community turnover. This was followed by drift and other processes (8.33% to 19.44%) and homogeneous dispersal (1.39% to 15.28%) (Figure 5B).

**Figure 6:**
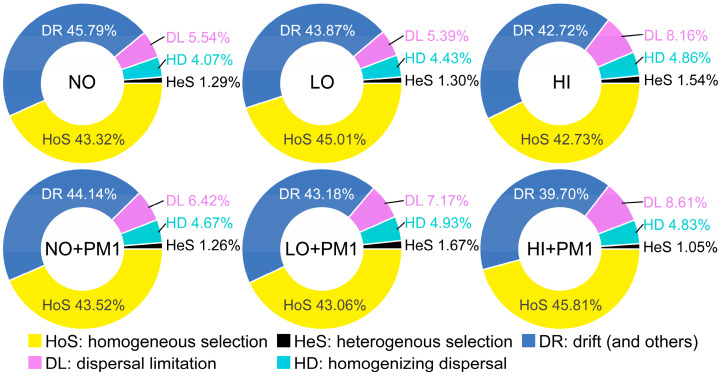
The relative importance of different ecological processes based on iCAMP analysis. NO: non-treatment soil, LO: low-dose selenium-contaminated soil, HI: high-dose selenium-contaminated soil, PM1: non-treatment soil + strain PM1, LO + PM1: low-dose selenium-contaminated soil + strain PM1, HI + PM1: high-dose selenium-contaminated soil + strain PM1.

Finally, the infer community assembly mechanisms by phylogenetic-bin-based null model analysis (iCAMP) was used to quantify the response of soil bacterial community assembly to sodium selenite treatment and strain PM1. The iCAMP results indicated that homogeneous selection (HoS) and drift (DR) were predominant in bacterial community assembly compared to dispersal limitation (DL), heterogeneous selection (HeS), and homogenizing dispersal (HD), with their average importance being 42.3–45.81% and 39.70–45.79%, respectively (Figure 6). Stochastic processes (sum of DR, DL, and HD) explained a higher proportion of soil bacterial community variation than deterministic processes, supporting the results of the NST and NCM. Overall, the concentration of sodium selenite reduced the relative importance of DR and HoS, suggesting that under high sodium selenite conditions, environmental filtering and physical barriers have a more significant impact on microbial community structure. In the strain PM1 treatment group, DR increased while HoS decreased, indicating that the selective pressure of PM1 on soil bacteria gradually increased with the concentration of sodium selenite. After sodium selenite and strain PM1 treatment, the relative importance of HoS and DR slightly decreased, while the relative importance of DL and HD significantly increased, indicating a growing significance of stochastic processes in the sodium selenite and strain PM1 treatment groups (Figure 6).

## 4. Discussion

### 4.1. Effects of Sodium Selenite and Strain PM1 on Bacterial Diversity

This study reviews the effects of sodium selenite on soil bacterial community composition, diversity, and interrelationships. Focusing on the sodium selenite-degrading bacterium *R. qingshengii* strain PM1, we evaluated its role in the restoration of soil microbial communities. We also preliminarily explored the impact of sodium selenite and strain PM1 on the functional aspects of soil microbial communities. In our research, there was no significant difference in the alpha diversity of soil bacterial communities during the initial phase (10 days). However, the alpha diversity index of certain community types showed a significant decrease (*p* < 0.05) in the mid to late stages (20–30 days) (Figure 1).

Soil physicochemical properties are critical characteristics that play a crucial role in the aggregation of bacterial communities [45]. To assess the relative contributions of environmental parameters to microbial community variation, we evaluated soil properties, including soil nutrients and sodium selenite concentration, to identify the factors driving community change. The random forest and SEM analyses indicated that bacterial structure was influenced by the combined effects of strain PM1 and sodium selenite concentration. Sodium selenite increased the relative abundance of *Actinobacteria* while decreasing the relative abundance of *Acidobacteria* (Figure 1). Similarly, the relative abundance of *Actinobacteria* also increases in heavy metal-contaminated soils [46,47,48]. Notably, *Actinobacteria* are widely distributed bacteria that exhibit adaptability to exogenous compounds in various environments [49]. The observed changes in *Actinobacteria* might be attributed to their rapid response to sodium selenite exposure. Additionally, heavy metal exposure has been reported to reduce the relative abundance of *Acidobacteria* and *Nitrospira* [50]. Members of the phylum *Acidobacteria* play essential roles in biogeochemical cycles, including those of carbon, iron, nitrogen, and hydrogen [51]. Therefore, exposure to sodium selenite can lead to an imbalance in bacterial community structure, potentially posing risks to soil microbial ecology.

Interestingly, both *Actinobacteria* and *Proteobacteria* dominate under selenium stress, likely due to their metal resistance mechanisms and their roles in crucial biogeochemical cycles such as nitrogen, carbon, and sulfur cycling. *Actinobacteria*, for instance, are involved in nitrogen fixation and organic matter decomposition, contributing to soil fertility and ecosystem functioning [52]. Proteobacteria, known for their diverse metabolic capabilities, are instrumental in reducing toxic compounds and participating in carbon and nitrogen cycling [53,54]. Furthermore, both bacterial phyla play a significant role in selenium detoxification [55], particularly through mechanisms like selenium reduction to less toxic forms, such as Se⁰ and organic selenium compounds (e.g., selenomethionine) [12]. The increased abundance of these bacteria under selenium stress suggests that they not only help mitigate selenium toxicity but also enhance nutrient cycling and organic matter decomposition, thus potentially improving soil health and ecosystem resilience in selenium-contaminated environments.

The dynamic analysis of sodium selenite residue indicates that soil inoculated with strain PM1 can significantly enhance the removal of sodium selenite, with degradation rates exceeding 63% for both high and low doses within 30 days (Figure 1A). Therefore, inoculation with strain PM1 could be beneficial for the remediation of sodium selenite-contaminated soils. To the best of our knowledge, this is the first report highlighting the promising bioremediation potential of strain PM1 in sodium selenite-contaminated soils. However, while these laboratory results are encouraging, the real-world applicability of this bioremediation strategy requires further investigation, as laboratory conditions may not fully replicate the complexities of field environments. To address this, we will discuss the challenges of scaling bioremediation in the field, including factors such as soil heterogeneity, microbial community interactions, and environmental variability. Therefore, we propose future studies that incorporate field data, such as field trials or microcosm experiments, to assess the practical applicability of *R. qingshengii* PM1 in real-world bioremediation scenarios.

Many studies have shown that a decline in microbial diversity can lead to the degradation of soil ecosystem functions [56], particularly in the context of heavy metal contamination [57]. A reduction in diversity typically results in the loss of functional redundancy, where a few microbial communities replace multiple species with overlapping functions, potentially affecting key ecological processes in the soil, such as nutrient cycling, nitrogen fixation, organic matter decomposition, and pathogen suppression [58]. In selenium-contaminated soils, reduced microbial diversity may cause the loss of diverse microbial populations that can respond to environmental changes, thereby weakening the soil’s self-repair capacity and resilience. Moreover, a decrease in functional microbial communities could lead to reduced soil fertility, destabilize soil structure, and impair plant growth [59]. From a soil health perspective, microbial diversity is a crucial safeguard for maintaining soil ecological functions [60]. The diversity and complexity of microbial communities provide higher ecological resilience, helping soils cope with pollutants, climate change, and other environmental stresses [61]. Therefore, a reduction in alpha diversity is not only an indicator of changes in microbial species but could also serve as an early warning sign of declining soil health. Particularly in environments with high selenium contamination, functional changes in microbial communities may hinder the cycling of elements such as carbon and nitrogen, ultimately affecting the sustainability and ecological function of the soil.

### 4.2. Co-Occurrence Network Analysis of Soil Microbial Community

Microbes do not exist in isolation but form complex communities, which are crucial for maintaining stability in response to external disturbances [62]. In this study, we used a network based on random matrix theory to establish bacterial community co-occurrence patterns, revealing microbial interactions and responses to sodium selenite and strain PM1. The topological features of the network generally represent levels of interaction and connectivity [63]. Topological properties indicate that the nodes in the sodium selenite-treated bacterial community network had fewer connections but a higher average path distance (Table 3), suggesting that microbial communities respond more quickly to environmental changes [64]. This allows disturbances to spread immediately throughout the network, enabling changes in network structure and function [65].

As the concentration of sodium selenite increased, the number of links in the bacterial network gradually decreased (Table 3 and Figure 3), indicating that interspecies relationships are strengthened and stabilized in selenium-contaminated soil. In terms of modularity, the NO and NO + PM1 groups had notably fewer modules than the other four selenium-treated bacterial networks, indicating that the ecological functions of bacteria in selenium-treated soils are more complex. Based on the intermediate disturbance hypothesis [66], the soil microbial communities in the treatment groups remained relatively stable, with stronger potential relationships between nodes and higher modularity. This study found that exposure to sodium selenite reduced the complexity of the bacterial co-occurrence network. Similar studies have also reported that microbial network complexity decreased with increasing heavy metal concentrations [18,32]. A decrease in microbial network complexity, as observed with higher selenium concentrations, may lead to weaker microbial interactions and lower functional redundancy [58]. This reduction in redundancy can impair the soil ecosystem’s ability to recover from environmental disturbances, as fewer species or functional groups remain to maintain critical ecological functions [67]. Moreover, the simplification of microbial networks may disrupt key biogeochemical cycles, including nutrient cycling [68]. Specifically, selenium contamination may hinder the cycling of essential nutrients such as nitrogen, phosphorus, and carbon, which are mediated by diverse microbial communities [69]. As microbial interactions become more limited, the soil’s capacity for nutrient exchange, decomposition, and overall soil fertility could be compromised, affecting soil health and plant growth.

Nodes with high connectivity in the network are often designated as key taxa, playing an indispensable role in stabilizing the structure and function of the ecosystem [70]. We identified a total of 80 keystone OTUs under various treatment conditions to determine their different ecological functions (Figure 3 and Table S2). Many potential key OTUs belong to the phylum *Proteobacteria*, which are considered key taxa in bacterial communities [71]. Positive and negative correlations in networks may reflect species interactions, representing cooperation or niche overlap (positive) and competition or niche separation (negative) [72,73]. In our six bacterial networks, positive links outnumbered negative ones (Figure 3), reflecting the importance of microbial synergy, where most microbial species may cooperate to resist selenium contamination [74]. Additionally, microbial species competition can also stabilize community co-occurrence and promote network stability [75].

### 4.3. Stochastic and Deterministic Processes Structure Bacterial Community Assembly

The ecological processes of bacterial community assembly can reveal microbial community responses to selenite stress. The NST and iCAMP results showed that with increasing sodium selenite concentration, the contribution of stochastic processes first increased and then decreased, indicating that at low concentrations of sodium selenite, microbial community assembly was more driven by stochastic processes (Figure 5A). Similarly, previous studies indicated that stochasticity increases under low-stress or disturbance conditions [24]. Low disturbance can reduce the overall stress on the ecosystem, thereby weakening the expression of environmental filtering [76]. However, under high doses of sodium selenite, environmental stress may lead dominant bacterial groups to become more prevalent through selective pressure, reducing the contribution of stochastic processes. In the strain PM1 inoculation groups, the contribution of stochastic processes gradually decreased, indicating that strain PM1 helps enhance the role of selective processes, possibly by increasing the number and activity of bacteria beneficial to soil health, thus promoting microbial community recovery and stability.

The NST, NCM, and iCAMP results all support that stochastic processes dominated bacterial community assembly. Possible reasons include the following: First, the main members of all treatment groups were *Proteobacteria*, which have a broad ecological niche, and their community assembly was mainly driven by random collisions and colonization [77]. Second, sodium selenite may promote stochasticity. In this study, we found sodium selenite to be one of the most significant factors influencing microbial communities (Figure 2D,F). According to Dini-Andreote et al. (2015), when a community experiences balanced disturbances, the relative role of stochastic processes may increase due to reduced selective pressures [78]. This suggests that the relatively less environmental filtering helps microbial communities. Some related studies support our conclusion. Wang, et al. [79] found that under acidic or high antimony (Sb) concentration conditions, the community assembly of Sb-resistant microorganisms was primarily controlled by a stochastic process. Mao, et al. [80] also detected that a stochastic process dominated the community assembly under Pb-Zn contaminated sites. The NCM result indicated that species dispersal was more limited under high concentrations of sodium selenite, likely due to the toxic effect of high selenium on soil microbes, hindering microbial dispersal. Contrary to our results, Li, et al. [81] found the assembly of *Nitrospira* community was governed by stochastic and deterministic processes in low- and high-salinity soils, respectively. This may be because variable selection has a more significant effect on low-salinity communities, while dispersal limitation has a higher proportion in high-salinity community assemblies.

To understand specific ecological assembly processes, we assessed the relative contributions of dispersal limitation (DL), heterogeneous selection (HeS), homogenizing dispersal (HD), homogeneous selection (HoS), and drift (DR) [78]. We found that HoS, DR, and DL mainly govern the soil microbial community assembly (Figure 6). Similar results have been reported in other studies, suggesting that HoS and DL were more important in deterministic and stochastic processes, respectively, in most long-term monoculture agriculture ecosystems [82]. DR may cause fluctuations in bacterial abundance due to random population size changes, including immigration-emigration and microbial movement events [83]. The iCAMP results further confirmed that sodium selenite impacts soil microbial community assembly primarily through stochastic processes. DL alone is insufficient to cause spatial variation in community composition, but the limited biological exchange between local communities may promote ecological community composition differentiation through random population size changes [42]. Overall, our findings verified that the diversity of soil bacterial communities under different concentrations of sodium selenite treatment was dominated by stochastic rather than deterministic processes.

In our study, we used four different models to assess bacterial community assembly processes. The results showed that NST and Sloan’s neutral community model, as well as iCAMP, all indicated that stochastic processes played a more critical role in bacterial community assembly than deterministic processes. However, β-NTI indicated that deterministic processes were the primary factors affecting bacterial community assembly in all samples. This inconsistency may be due to the sensitivity of each model to different ecological processes, leading to varying interpretations of the same dataset. Despite the inconsistency in results from different models, they collectively reveal the importance of both stochastic and deterministic processes in bacterial community assembly. Combining the results of multiple models is necessary to comprehensively understand bacterial community assembly mechanisms and accurately interpret experimental data.

## 5. Conclusions

In conclusion, this study reveals that sodium selenite and the sodium selenite-degrading bacterium *R. qingshengii* strain PM1 significantly influence soil microbial community structure and diversity. Our findings demonstrate that exposure to sodium selenite initially led to reductions in microbial diversity and a shift in dominant bacterial groups, particularly an increase in *Actinobacteria* and a decrease in *Acidobacteria*. Co-occurrence network analysis showed that sodium selenite altered microbial interactions, with reduced network complexity reflecting an adaptive community reorganization under selenium stress. Additionally, ecological analysis through NST, iCAMP, and neutral community models indicated that bacterial community assembly was predominantly governed by stochastic processes. These insights underscore the potential of strain PM1 for the bioremediation of selenium-contaminated soils, offering promising prospects for environmental restoration and soil health management.

## Figures and Tables

**Table 1:** Soil physicochemical properties under different treatment conditions.

	Treatment	Days	NO	LO	HI	NO + PM1	LO + PM1	HI + PM1
Soil Index								
Total nitrogen (g/kg)		D1	0.987 ± 0.021 b	1.035 ± 0.05 ab	1.083 ± 0.025 a	1.015 ± 0.015 ab	1.005 ± 0.037 b	1.018 ± 0.059 ab
		D2	1.072 ± 0.022 b	1.009 ± 0.048 bc	1.007 ± 0.036 bc	0.967 ± 0.017 c	1.158 ± 0.058 a	1.024 ± 0.013 ab
		D3	1.144 ± 0.025 a	1.015 ± 0.028 b	1.025 ± 0.031 b	1.136 ± 0.074 a	1.115 ± 0.071 a	1.091 ± 0.016 ab
		D4	1.061 ± 0.033 ab	1.045 ± 0.069 ab	0.967 ± 0.016 b	1.129 ± 0.095 a	1.09 ± 0.081 ab	1.059 ± 0.052 ab
Total phosphorus (g/kg)		D1	0.119 ± 0.004 ab	0.114 ± 0.006 bc	0.121 ± 0.009 ab	0.112 ± 0.006 bc	0.105 ± 0.004 c	0.127 ± 0.005 a
		D2	0.127 ± 0.004 b	0.112 ± 0.012 bc	0.129 ± 0.005 b	0.101 ± 0.008 c	0.105 ± 0.016 c	0.145 ± 0.002 a
		D3	0.142 ± 0.006 a	0.125 ± 0.018 ab	0.126 ± 0.007 ab	0.115 ± 0.009 b	0.093 ± 0.008 c	0.136 ± 0.012 a
		D4	0.121 ± 0.001 bc	0.112 ± 0.015 bc	0.13 ± 0.007 ab	0.108 ± 0.016 b	0.088 ± 0.005 c	0.138 ± 0.002 a
Total potassium (g/kg)		D1	16.13 ± 0.714 a	16.537 ± 0.884 a	16.077 ± 0.667 a	16.286 ± 0.133 a	16.598 ± 0.316 a	16.515 ± 0.307 a
		D2	16.729 ± 1.011 a	16.457 ± 0.134 a	16.474 ± 0.696 a	15.807 ± 0.589 a	16.802 ± 0.93 a	14.843 ± 0.325 a
		D3	17.678 ± 0.576 ab	16.733 ± 0.719 a	16.396 ± 0.563 b	15.348 ± 0.793 b	16.139 ± 0.833 b	16.719 ± 0.119 ab
		D4	16.34 ± 0.647 ab	16.304 ± 0.264 b	16.469 ± 0.059 ab	16.241 ± 1.288 b	16.433 ± 1.071 ab	16.843 ± 0.143 a
soil organic carbon (g/kg)		D1	23.428 ± 0.397 bc	26.641 ± 1.123 a	22.033 ± 0.566 bcd	19.958 ± 2.856 d	20.913 ± 1.767 cd	23.962 ± 1.45 ab
		D2	22.313 ± 0.791 a	17.947 ± 1.826 b	21.847 ± 0.966 a	17.776 ± 1.529 b	21.963 ± 0.474 a	24.038 ± 0.927 a
		D3	21.427 ± 0.356 b	22.289 ± 2.214 b	24.238 ± 0.746 b	20.984 ± 2.001 b	22.369 ± 2.449 b	25.79 ± 0.407 a
		D4	22.092 ± 0.624 ab	20.547 ± 1.492 b	20.769 ± 0.658 b	20.129 ± 0.664 b	20.217 ± 1.656 b	24.182 ± 0.662 a
FDA/(μg/h/g)		D1	173.291 ± 0.088 a	162.707 ± 8.967 a	120.756 ± 35.691 b	153.531 ± 6.618 a	153.377 ± 8.24 a	158.947 ± 4.007 a
		D2	172.654 ± 1.516 a	165.89 ± 9.985 a	169.639 ± 1.836 a	165.329 ± 22.543 a	163.896 ± 11.913 a	178.88 ± 18.85 a
		D3	171.225 ± 8.035 b	212.815 ± 27.198 a	180.88 ± 24.174 b	164.434 ± 14.076 b	226.992 ± 32.313 a	217.729 ± 19.977 a
		D4	162.35 ± 11.481 a	159.038 ± 11.327 a	177.519 ± 17.6 a	176.987 ± 10.692 a	173.412 ± 4.087 a	170.983 ± 3.82 a
S-PPO/(nmol/h/g)		D1	109.295 ± 7.801 c	113.981 ± 19.643 c	147.156 ± 29.89 bc	148.464 ± 32.805 c	170.505 ± 30.599 ab	197.903 ± 8.109 a
		D2	136.916 ± 17.742 e	175.618 ± 15.708 de	217.919 ± 13.38 a	173.438 ± 16.539 cd	181.822 ± 10.13 b	176.932 ± 11.727 bc
		D3	126.319 ± 28.051 b	161.912 ± 21.256 b	188.604 ± 9.14 ab	184.329 ± 16.739 ab	166.742 ± 36.842 ab	200.646 ± 17.903 a
		D4	128.488 ± 20.969 b	148.404 ± 34.088 b	171.425 ± 32.597 b	182.622 ± 12.884 ab	188.878 ± 19.867 ab	213.576 ± 2.693 a
S-UE/(μg/d/g)		D1	806.489 ± 73.395 a	795.134 ± 20.826 a	703.71 ± 34.947 bc	688.611 ± 25.073 ab	559.802 ± 17.308 d	658.454 ± 78.084 c
		D2	771.215 ± 88.486 a	696.542 ± 35.015 ab	680.084 ± 86.636 b	776.618 ± 80.959 ab	618.726 ± 50.393 b	662.824 ± 80.702 b
		D3	764.065 ± 29.394 a	636.677 ± 40.324 b	697.946 ± 67.998 b	741.54 ± 43.484 ab	641.453 ± 90.763 b	682.97 ± 25.777 b
		D4	762.441 ± 28.119 a	709.149 ± 23.83 a	713 ± 20.289 ab	749.958 ± 50.421 a	484.428 ± 161.467 c	594.139 ± 52.838 bc
S-β-GC/(nmol/h/g)		D1	466 ± 6.478 b	544.962 ± 56.584 a	508.089 ± 13.26 b	498.353 ± 101.958 ab	414.989 ± 33.007 bc	456.106 ± 68.039 b
		D2	472.979 ± 28.44 b	569.823 ± 64.04 a	503.783 ± 18.627 a	522.101 ± 77.591 a	474.589 ± 8.473 b	582.424 ± 42.806 a
		D3	535.116 ± 12.984 ab	446.291 ± 159.617 a	405.794 ± 9.023 b	488.613 ± 19.56 ab	436.045 ± 84.672 ab	481.554 ± 63.823 ab
		D4	504.005 ± 47.579 b	497.086 ± 14.369 a	439.021 ± 48.118 b	440.767 ± 8.753 b	419.026 ± 28.101 b	440.601 ± 50.444 b
S-NR/(μmol/d/g)		D1	8.111 ± 1.586 b	25.303 ± 10.171 a	4.374 ± 12.49 c	3.987 ± 0.145 c	6.319 ± 0.592 bc	5.151 ± 3.338 bc
		D2	6.376 ± 3.402 ab	9.254 ± 1.708 a	6.19 ± 5.41 ab	2.516 ± 1.702 b	6.167 ± 1.157 ab	3.493 ± 2.578 b
		D3	2.733 ± 0.913 bc	7.245 ± 3.106 a	1.118 ± 3.749 d	1.877 ± 0.537 cd	3.789 ± 1.181 a	1.874 ± 1.618 cd
		D4	2.723 ± 1.724 b	3.996 ± 0.338 b	7.07 ± 1.133 a	2.197 ± 2.906 b	2.839 ± 0.757 b	2.797 ± 2.004 b
S-POD/(nmol/h/g)		D1	493.4 ± 20.718 a	470.877 ± 39.774 ab	418.756 ± 69.792 ab	399.856 ± 10.232 b	368.075 ± 11.153 b	311.197 ± 21.661 c
		D2	472.452 ± 35.048 a	431.043 ± 15.6 ab	419.168 ± 19.954 bc	417.587 ± 22.441 bc	396.07 ± 26.195 c	395.677 ± 9.571 c
		D3	480.793 ± 11.427 a	461.878 ± 14.039 ab	442.632 ± 18.529 ab	424.504 ± 15.357 b	388.964 ± 12.348 c	468.753 ± 37.01 ab
		D4	511.5 ± 17.425 a	504.417 ± 15.747 a	449.667 ± 44.153 ab	448.603 ± 12.402 b	437.76 ± 18.726 b	462.54 ± 38.444 b

Data are presented as mean ± standard deviation, with different letters indicating statistically significant differences between treatments at *p* < 0.05.

**Table 2:** Significance tests of the effects of sodium selenite, strain PM1, and incubation time on the bacterial community with three different statistical approaches.

	Adonis		ANOSIM		MRPP	
	*F*	*P*	*R*	*P*	*δ*	*F*
Incubation time	4.761	0.001	0.2976	0.001	0.313	0.001
Strain PM1	7.0069	0.001	0.2498	0.001	0.3246	0.001
Different dose of Na_2_SeO_3_	3.987	0.001	0.1876	0.001	0.3252	0.001

**Table 3:** Topological properties of empirical networks of different bacterial communities and their associated random ecological networks.

Network Name	Topological Properties	NO	LO	HI	NO + PM1	LO + PM1	HI + PM1
Empirical	Similarity threshold	0.89	0.89	0.89	0.89	0.89	0.89
	Total nodes	658	736	605	700	665	657
	Total links	981	1504	660	1579	1030	919
	Average degree (avgk)	2.982	4.087	2.182	4.511	3.098	2.798
	Centralization of degree (CD)	0.063	0.057	0.02	0.061	0.045	0.028
	Average path distance (GD)	6.081	7.323	7.516	5.873	4.966	7.766
	Average clustering coefficient (avgCC)	0.084	0.129	0.075	0.131	0.083	0.089
	Centralization Betweenness (CB)	0.076	0.128	0.074	0.067	0.029	0.071
	Modularity	0.64	0.64	0.84	0.664	0.703	0.817
Random networks	Modularity	0.613 ± 0.005	0.474 ± 0.005	0.779 ± 0.007	0.441 ± 0.004	0.587 ± 0.005	0.654 ± 0.005
	Average path distance (GD)	4.414 ± 0.062	3.897 ± 0.037	5.860 ± 0.147	3.785 ± 0.034	4.222 ± 0.061	4.918 ± 0.054
	Average clustering coefficient (avgCC)	0.0.14 ± 0.003	0.029 ± 0.004	0.003 ± 0.002	0.032 ± 0.004	0.018 ± 0.004	0.007 ± 0.002

## Data Availability

The original contributions presented in this study are included in the article/Supplementary Materials. Further inquiries can be directed to the corresponding author.

## Supplementary Materials

The following supporting information can be downloaded at: https://www.mdpi.com/article/10.3390/microorganisms12122458/s1. Figure S1 Good’s coverage curves of soil bacterial communities across all treatments; Figure S2 Pearson correlations between network complexity and sodium selenite concentration, stability indices under warming; Table S1 Sequencing Data Summary for Soil Bacterial Communities Across All Samples; Table S2 The keystone OTU in each co-occurrence network; Table S3 The normalized stochasticity ratio (NST) in different bacterial communities; Table S4 The neutral community model (NCM) in different bacterial communities.
