# Supplementary material for: Bioremediation Potential of Rhodococcus qingshengii PM1 in Sodium Selenite-Contaminated Soil and Its Impact on Microbial Community Assembly
Source: Microorganisms. 2024 Nov 29;12(12):2458. doi: 10.3390/microorganisms12122458 (PMC11677749; doi:10.3390/microorganisms12122458)
Supplement: Supplementary file 1 [file microorganisms-12-02458-s001.zip › Fig. S2.pdf]

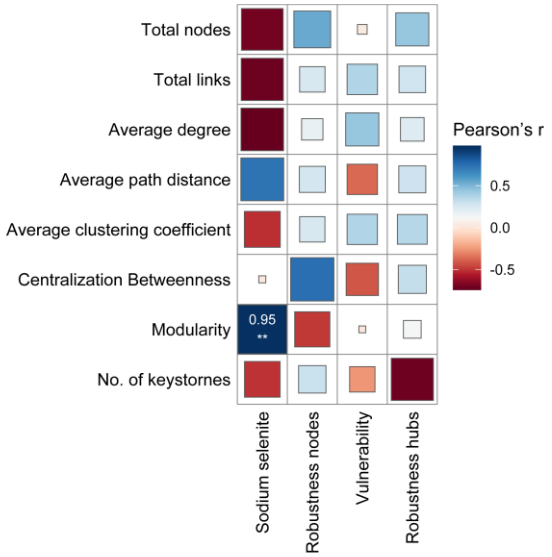

Fig. S2 Pearson correlations between network complexity and sodium selenite concentration, stability indices under warming
