# Supplementary material for: Bioremediation Potential of Rhodococcus qingshengii PM1 in Sodium Selenite-Contaminated Soil and Its Impact on Microbial Community Assembly
Source: Microorganisms. 2024 Nov 29;12(12):2458. doi: 10.3390/microorganisms12122458 (PMC11677749; doi:10.3390/microorganisms12122458)
Supplement: Supplementary file 1 [file microorganisms-12-02458-s001.zip › Table S3.pdf]

Table S3 The normalized stochasticity ratio (NST) in different bacterial communities

Treatments	Mean	SD
NO	0.838361	0.020494
LO	0.867752	0.023287
HI	0.825829	0.024827
NO+PM1	0.850571	0.025397
LO+PM1	0.807934	0.03116
HI+PM1	0.815905	0.023331
