# Supplementary material for: Bioremediation Potential of Rhodococcus qingshengii PM1 in Sodium Selenite-Contaminated Soil and Its Impact on Microbial Community Assembly
Source: Microorganisms. 2024 Nov 29;12(12):2458. doi: 10.3390/microorganisms12122458 (PMC11677749; doi:10.3390/microorganisms12122458)
Supplement: Supplementary file 1 [file microorganisms-12-02458-s001.zip › Table S4.pdf]

Table S4 The neutral community model (NCM) in different bacterial communities

	R^2	*N*	*m*	*Nm*
All	0.8798	33599	0.9041	30378.3717
NO	0.826	33599	1.0591	35584.0937
LO	0.8314	33599	1.0043	33744.9574
HI	0.8281	33599	1.0078	33862.7201
NO+PM1	0.8258	33599	1.0285	34555.7792
LO+PM1	0.8338	33599	1.0419	35007.8896
HI+PM1	0.8242	33599	0.943	31682.562
