# Supplementary figures and images for: Bioremediation Potential of Rhodococcus qingshengii PM1 in Sodium Selenite-Contaminated Soil and Its Impact on Microbial Community Assembly
Source: Microorganisms. 2024 Nov 29;12(12):2458. doi: 10.3390/microorganisms12122458 (PMC11677749; doi:10.3390/microorganisms12122458)

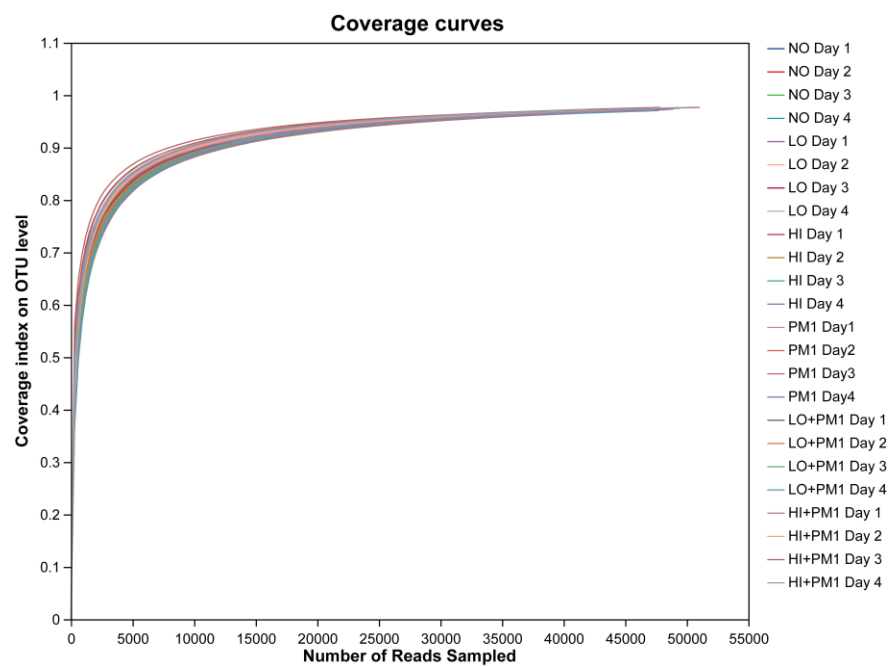

Fig. S1 Good's coverage curves of soil bacterial communities across all treatments

Supplement: Supplementary file 1 [file microorganisms-12-02458-s001.zip › Fig. S1.pdf]
